# Prospective Evaluation of Ocular Anterior Segment Morphology Changes in the Steep Trendelenburg Position During Robotic-Assisted Laparoscopic Prostatectomy

**DOI:** 10.3390/jcm15020731

**Published:** 2026-01-16

**Authors:** Mototaka Sato, Eisuke Shimizu, Atsuki Matsukawa, Ryoya Mizuno, Satoshi Kamido, Takahiro Mizukami, Norichika Ueda, Yoko Fujimoto, Norihide Tei, Osamu Miyake

**Affiliations:** 1 Department of Urology, Toyonaka Municipal Hospital, Toyonaka 5608565, Osaka, Japan; mototaka1022@yahoo.co.jp (M.S.); atsuki.mattsu@gmail.com (A.M.); mizunotebyppmr@gmail.com (R.M.); drnorick@yahoo.co.jp (N.T.); yoka495@yahoo.co.jp (O.M.); 2 Department of Ophthalmology, Keio University School of Medicine, Shinjyuku-ku, Tokyo 1608582, Japan; ophthalmolog1st.acek39@keio.jp; 3 OUI Inc., Minato-ku, Tokyo 1070062, Japan; 4 Medical Center for Translational Research, Department of Medical Innovation, Osaka University Hospital, Suita 5650871, Osaka, Japan; s.kamido@gmail.com; 5 Fuchu Eye Center, Fuchu Hospital, Izumi 5940076, Osaka, Japan; 6 Department of Urology, Osaka University Graduate School of Medicine, Suita 5650871, Osaka, Japan; ueda@uro.med.osaka-u.ac.jp; 7 Fujimoto Ophthalmic Clinic, Osaka 5460013, Osaka, Japan; f-yoko-f@dg7.so-net.ne.jp

**Keywords:** robot-assisted laparoscopic prostatectomy, steep Trendelenburg position, CO_2_ pneumoperitoneum, anterior chamber depth, Smart Eye Camera, smart ophthalmology

## Abstract

**Background/Objectives**: Surgery performed in the steep Trendelenburg position is considered potentially detrimental to ocular structures. This study aimed to evaluate morphological changes in the anterior segment during robot-assisted laparoscopic prostatectomy (RALP). **Methods**: This was a single-center, prospective observational study involving 60 eyes of 30 consecutive patients undergoing RALP between May and November 2021. Anterior segment images were obtained using a Smart Eye Camera before surgery (supine and awake), during surgery (supine and steep Trendelenburg under anesthesia), and after surgery (supine). Assessed parameters included the iridocorneal angle, the ratio of peripheral anterior chamber depth to peripheral corneal thickness based on Van Herick Plus grading, and pupil diameter. Each parameter in the steep Trendelenburg position under anesthesia was compared with measurements obtained in the supine position under anesthesia and in the awake condition. The primary outcome was the comparison of anterior segment morphological changes between the supine and steep Trendelenburg positions during RALP. **Results**: Upon transition to the steep Trendelenburg position, anterior chamber depth significantly decreased (*p* < 0.001), recovering after returning to the supine position. The proportion of eyes classified as having narrowed anterior chambers increased significantly (*p* < 0.001), with more than 60% showing shallower chambers relative to preoperative measurements. Extreme anterior chamber narrowing occurred in 3 out of 410 intraoperative assessments (1%). Pupils were uniformly constricted under anesthesia. **Conclusions**: Steep Trendelenburg positioning significantly reduces anterior chamber depth. This morphological alteration may contribute to the marked increase in intraocular pressure observed during RALP.

## 1. Introduction

Prostate cancer (PCa) accounts for 7.3% of all cancers worldwide and is the second most common malignancy in men, with incidence increasing as the global population ages [1,2,3]. Approximately half of all patients with PCa undergo radical prostatectomy [4]. Robot-assisted laparoscopic prostatectomy (RALP) is an advanced surgical technique widely performed worldwide for patients with localized PCa [5]. However, concerns have been raised about the potential adverse effects of RALP on ocular health, particularly regarding an elevation in intraoperative intraocular pressure (IOP). Elevated IOP can lead to ocular complications such as postoperative visual loss [6]. IOP is regulated by aqueous humor dynamics; obstruction of aqueous humor outflow during surgery may lead to an elevation in intraoperative IOP. During RALP, such elevation has been attributed to choroidal distention caused by venous congestion resulting from both carbon dioxide (CO_2_) pneumoperitoneum and/or the steep Trendelenburg position (STP) [7]. CO_2_ pneumoperitoneum leads to decreased venous drainage due to the increase in central venous pressure and arterial partial pressure of CO_2_ (pCO_2_) [7,8]. Choroidal blood flow is reported to increase by 3.6% for every 1 mmHg increase in pCO_2_ [9]. The cephalad fluid shift due to STP similarly contributes to choroidal distention [7,8,9]. Theoretically, nearly all patients undergoing RALP with both CO_2_ pneumoperitoneum and STP might develop elevated IOP.

IOP has been demonstrated to increase during RALP in all studies conducted thus far [7,8,9,10,11,12,13,14,15,16,17,18,19,20,21]. IOP slightly decreases upon induction of general anesthesia compared with preoperative measurements [7,8,10,11,17]; however, it slightly increases upon the initiation of CO_2_ pneumoperitoneum, significantly rises with the commencement of the STP, and gradually increases over time during both CO_2_ pneumoperitoneum and STP. IOP decreases upon returning to the supine position, and postoperatively, it is nearly equivalent to preoperative levels [7,8,9,12,16,17,18,19,20]. However, few studies have reported ocular dysfunction after RALP [7,10,13,14,15]. The elevation in IOP during RALP is evident and deemed acceptable.

Conversely, several cases of large IOP increases during RALP have been reported [8,9], and a minimal risk of postoperative blindness exists. In 2007, a case of ischemic optic neuropathy was reported by Weber et al. [22], and other cases of subclinical visual field abnormalities have since been reported after RALP [7,13]. Despite the possibility that an unacceptable IOP increase may have occurred in these cases, the causative factors for the rapid IOP increase during RALP remain largely unidentified and unpredictable.

The morphology of the anterior segment serves as a critical parameter for predicting the risk of IOP elevation, and shallow anterior chambers are a known risk factor for angle-closure glaucoma [23]. Eyes with an average anterior chamber depth (ACD) of less than 1.9 mm can develop angle closure [24]. Recently, a case of bilateral acute angle-closure glaucoma following general anesthesia has been reported [25]. Typically, detailed observation of the anterior segment is conducted primarily with a slit lamp microscope in ophthalmological settings, making in-surgery evaluations challenging. The Smart Eye Camera (SEC), developed by Shimizu et al., functions as an iPhone attachment that enables precise observation of the anterior segment of the eye in any clinical setting [26,27]. A previous study has demonstrated that the SEC can estimate ACD with a level of accuracy comparable to that of conventional anterior segment optical coherence tomography [28]. To the best of our knowledge, no reports have focused on the morphological changes in the anterior segment during robot-assisted pelvic surgeries, including RALP. In this preliminary study, we used the SEC to examine anterior segment morphological changes predisposing eyes to IOP elevation during RALP.

## 2. Materials and Methods

### 2.1. Study Population

This study was designed as a single-center, prospective, observational investigation; consecutive patients with PCa scheduled for RALP at Toyonaka Municipal Hospital between 12 May 2021 and 11 November 2021 were recruited. To investigate the impact of the RALP environment on the eyes, we excluded men diagnosed with PCa who had a history of angle-closure glaucoma, an eye with an intraocular lens insertion, current or past ocular trauma, a body mass index of >30 kg/m^2^, or uncontrolled hypertension. This study was conducted in compliance with the Declaration of Helsinki and received approval from the Ethical Review Committee of the institution (IRB number 202010058). Written informed consent was acquired from all participating patients.

### 2.2. Robot-Assisted Laparoscopic Prostatectomy

RALP was performed for localized PCa using the intraperitoneal approach with the da Vinci Surgical System Xi (Intuitive Surgical, Sunnyvale, CA, USA) by four urologists, including one proctor. After port placement in the supine position, patients were tilted to a 25° steep Trendelenburg position (STP) for the robotic procedure and returned to supine for tissue removal and closure. Pneumoperitoneum was maintained with CO_2_ at 10 mmHg, temporarily increased to 15 mmHg during dorsal vein complex ligation. All surgeries were conducted in the morning or early afternoon to minimize diurnal variations in ocular parameters.

Following adequate oxygenation, rapid intravenous administration of propofol (1.0–2.0 mg/kg) and rocuronium (1.0 mg/kg) was initiated, followed by endotracheal intubation. Intraoperative anesthesia was maintained with desflurane (4–6%), remifentanil (0.1–0.3 μg/kg/min), and fentanyl (3–4 μg/kg), along with rocuronium (10 mg/h) for maintaining a stable muscle relaxation effect. Sugammadex sodium (2–4 mg/kg) was used for reversing muscle relaxation. Patients were mechanically ventilated to maintain appropriate levels of oxygen saturation (SpO_2_) and end-tidal CO_2_ (ETCO_2_).

### 2.3. Smart Eye Camera

Utilization of the SEC (SLM-i07/SLM-i08SE, OUI Inc., Tokyo, Japan; 13B2X10198030101/13B2X10198030201) by trained urologists facilitated video capture of the anterior segment of each patient’s eyes at predetermined intervals using an iPhone 7 (Apple Inc., Cupertino, CA, USA). Anterior segment photographs were taken in the awake, supine position at the urology outpatient clinic before surgery (Tpre) and in the operating room immediately after anesthesia induction (Tanesth). During RALP, imaging was performed immediately after adopting the steep Trendelenburg position (T0), every hour thereafter (T1–T5), after returning to supine (Tsupine), and at surgery completion (Tpostop). On the following day, images were obtained again in the awake, supine position in the hospital room (Tpost).

**Figure 1:**
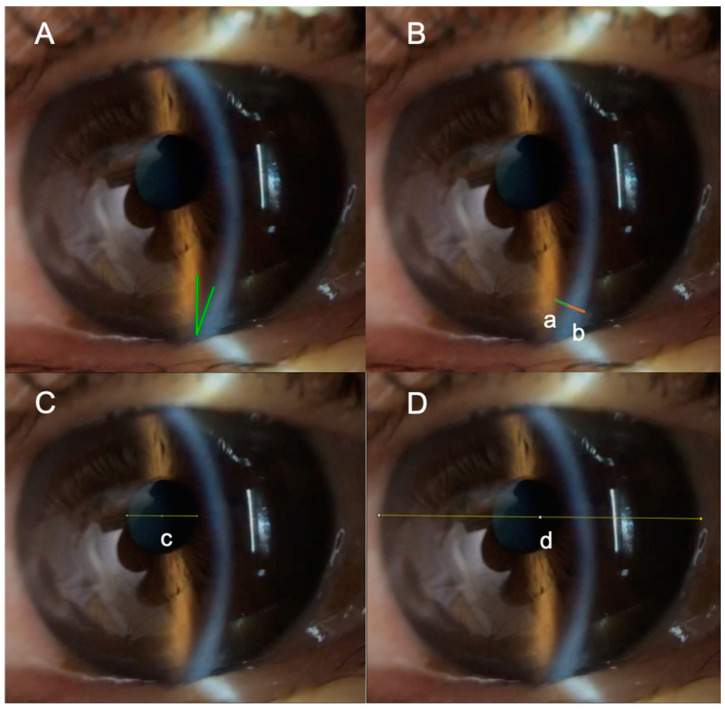
Evaluation of the anterior segment of the eye using SEC. The illumination angle of the microscope is set at 30°, and the objective lens magnification is 40×. The brightest, narrowest, 3 mm high vertical beam of light is directed across the lower eyelid margin at the 6 o’clock position, with part of it overlying the adjacent sclera. (**A**) ICA: Angle between the corneal endothelium and the anterior surface of the iris at the sclerolimbal junction, corresponding to the angle formed by the two green reference lines. (**B**) The PAC:PCT ratio was evaluated at the most peripheral area where the anterior chamber was clearly visible (a/b). (**C**,**D**) PD and CD were measured at the site of their maximum diameters, and the PD:CD ratio was evaluated (c/d). SEC, smart eye camera; ICA, iridocorneal angle; PAC, peripheral anterior chamber; PCT, peripheral corneal thickness; PD, pupil diameter; CD, corneal diameter.

We sent the captured video images to the Department of Ophthalmology, Keio University School of Medicine, where still images for analysis were selected, and evaluations were conducted by an ophthalmologist (E.S.) and another physician (S.K.), blinded to the patients’ clinical outcomes. Using a short, vertical slit beam not reaching the pupil, the anterior segment was observed. The inferior angle at the sclerolimbal junction at the 6 o’clock position was evaluated by measuring the iridocorneal angle (ICA) (Figure 1A) and the ratio of peripheral anterior chamber depth to peripheral corneal thickness (PAC:PCT) (Figure 1B). The iridocorneal angle width (in degrees) was categorized into four groups: <15° (occludable/narrow angle), 15–20° (moderately narrow), 20–30° (intermediate), and >30° (wide). The cutoffs of 15°, 20° and 30° were chosen based on previous imaging studies that have used similar thresholds to define occludable angles and to stratify angle width [29,30,31]. The PAC:PCT ratio was calculated and stratified using Van Herick Plus (VHp) grading, based on which the participants were classified into four groups: I (<1/4), II (1/4–1/2), III (>1/2–1), and IV (>1) [32]. The maximum pupil diameter (PD) was evaluated as a ratio to the maximum corneal diameter (CD) (Figure 1C,D). The images were analyzed ImageJ (V.1.50i), with ICA measurements taken using the angle tool, and assessments of ACD, corneal thickness, CD, and PD conducted using a straight-line tool. The ICA was defined as the angle formed between two lines originating at the sclerolimbal junction: one drawn along the corneal endothelium and the other along the anterior surface of the iris [32]. These anatomical landmarks were consistently identified across all images. Image quality was assessed prior to analysis, and images with obscured anatomical landmarks, poor focus, motion artifacts, or significant conjunctival edema that prevented accurate identification of the corneal endothelium or anterior iris surface were excluded. The selection process followed predefined criteria to minimize selection bias (Supplementary Figure S1). All images were derived from the original iPhone video recordings, which were captured in 1080p HD resolution (1920 × 1080 pixels), providing consistent image quality for angular measurements. The SEC has been validated in prior studies showing good agreement with conventional anterior segment optical coherence tomography in assessing anterior chamber structures, supporting its suitability as a practical imaging modality in perioperative settings where standard anterior segment optical coherence tomography may not be feasible [27,28].

### 2.4. Statistical Analysis

Since this was a pilot study, no prior sample size design was performed. Considering the feasibility, the maximum number of participants that could be enrolled during the six-month study period was used as the sample size. The post hoc power of ICA for Tanesth and T2 with the actual collected sample size was 95.6%. Inter-rater agreement for the VHp score, an ordinal categorical variable, was evaluated using Cohen’s kappa statistic. Agreement for the continuous measures (ICA and the PD:CD ratio) was assessed using the intraclass correlation coefficient from a two-way random-effects model, single measurement (Intraclass Correlation Coefficients (ICC) [2, 1]). Participant characteristics were summarized using frequency distributions (counts, percentages) and descriptive statistics (mean, standard deviation, range). The distributions of the primary endpoints—mean ICA and the PD:CD ratio—were examined using histograms to confirm that they could be treated as approximately normal. In addition, ICA was categorized into four groups (>30°, 20–30°, 15–20°, <15°) and summarized as an ordinal variable. Descriptive statistics were computed at each measurement time point and, further, multiple time points were aggregated by condition as follows: “Awake, supine” = Tpre + Tpost; “Under anesthesia, supine” = Tanesth + Tsupine + Tpostop; “Under anesthesia, STP” = T0 + T1 + T2 + T3. Comparisons of ICA and VHp scores across conditions were performed using ordinal logistic mixed-effects models. A random effect for subject was included to account for multiple eyes per subject and for the inclusion of the same subject’s data when aggregating by condition. Results are presented as odds ratios (ORs) with 95% confidence intervals (CIs) and *p*-values, using “Awake, supine” and “Under anesthesia, supine” as the reference conditions, respectively. For each eye, frequency tabulations were produced for changes from Tpre in ICA and VHp score. These tabulations were stratified by the initial ICA value and initial VHp score, and overall counts were provided for eyes showing decreases versus no change. Finally, predictors of ICA < 20° at T2 were explored using a mixed-effects logistic regression model with a random subject effect. Because only three eyes met the endpoint (ICA < 20° at T2), analyses were limited to univariable models. Candidate predictors included age (baseline characteristic), ICA at T1, and mean arterial pressure (MAP), ETCO_2_, peak inspiratory pressure (PIP), and infusion volume at T1 and at T2. All statistical tests were two-sided with a significance level of 0.05. Analyses were conducted using SPSS 29.0 for Windows (IBM Japan, Ltd., Tokyo, Japan).

## 3. Results

### 3.1. Patients’ Characteristics

In total, 60 eyes from 30 consecutive men scheduled for RALP were included. Three eyes belonging to two men with intraocular lens insertion and no preoperative declaration were excluded. A total of 471 videos from 57 eyes of 29 men were recorded. Fifty-four videos were omitted due to upload failure or inadequate image quality. During RALP, a few cases required more than 4 h in STP, and the number of T4 and T5 videos was very small (7), and were excluded from the analysis, culminating in the inclusion of 410 videos in the final analysis (Supplementary Figure S1). Patient demographics are summarized in Table 1. The mean age was 69.6 years (range: 57–79). Three patients (10.3%) had bilateral normal tension open-angle glaucoma. The mean durations for CO_2_ pneumoperitoneum and STP were 224.4 min (range: 98–337) and 215.3 min (range: 88–316), respectively.

### 3.2. ICA Values, VHp Score, and PD:CD Ratio at Various Time Points

**Figure 2:**
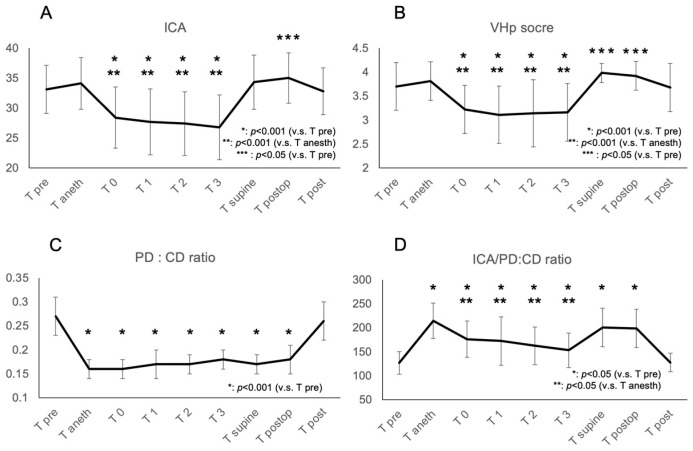
Morphological changes in the anterior segment during RALP. (**A**) Mean ICA values for each time point. Error bars represent SD. (**B**) Mean VHp score for each time point. Error bars represent SD. (**C**) Mean PD:CD ratio for each time point. Error bars represent SD. (**D**) Mean ICA/PD:CD ratio for each time point. Error bars represent SD. RALP, robot-assisted laparoscopic prostatectomy; SD, standard deviation; VHp, Van Herick plus grading.

The VHp score assessed by the two evaluators (E.S. and S.K.) demonstrated a high level of agreement (kappa coefficient, 0.819) (Supplementary Table S1). Similarly, ICA and PD:CD ratio also showed high agreement, with intraclass correlation coefficients of 0.931 and 0.946, respectively (Supplementary Table S2). Mean ICA values, VHp Score, and PD:CD ratio at various time points are listed in Table 2. Following the transition to STP (T0-T3), mean ICA values exhibited a significant decrease compared with both Tpre and Tanesth (*p* < 0.001 for both), returning to baseline levels upon resuming the supine position, and the ICA values at Tpostop were significantly higher than at Tpre (*p* < 0.05) (Figure 2A). After transitioning to STP (T0-T3), the mean VHp score showed a significant decrease relative to both Tpre and Tanesth (*p* < 0.001 for both). After returning to the supine position, the ACD increased, and the VHp scores at Tsupine and Tpostop were significantly higher than at Tpre (*p* < 0.05) (Figure 2B). Under general anesthesia (Tanesth to Tpostop), pupil diameter displayed significant and uniform constriction compared with awareness condition measurements (*p* < 0.001) (Figure 2C). We examined the ICA/PD:CD ratio across timepoints (Figure 2D). Compared with the awake state, the ratio was significantly higher under anesthesia. Within the anesthetized condition, the ratio was significantly lower in STP than in the supine position and improved upon returning to the supine position. Due to the significant difference in pupil diameter, interpreting the difference in the ICA/PD:CD ratio between the awake and anesthetized states is difficult. Under general anesthesia, positional changes significantly affected the iridocorneal angle.

Table 3 shows ICA values recorded at each measurement time point for all eyes. Notably, no narrow anterior chamber configurations were noted preoperatively (ICA 15–20°: 0 [0%], ICA < 15°: 0 [0%]). Compared with the “awake, supine” and “under anesthesia, supine” conditions, the ICA values were significantly lower during the “under anesthesia, STP” condition (OR = 0.050, 95% CI: 0.025–0.101, *p* < 0.001; OR = 0.033, 95% CI: 0.016–0.067, *p* < 0.001, respectively) (Table 4).

Table 5 shows VHp scores recorded at each measurement time point for all eyes. Similarly, no narrow anterior chamber configurations were noted preoperatively (VHp score 2: 0 [0%]; VHp score 1: 0 [0%]). We found that the VHp score during the “under anesthesia, STP” condition was significantly lower compared with both the “awake, supine” and “under anesthesia, supine” conditions (OR = 0.048, 95% CI: 0.023–0.100, *p* < 0.001; OR = 0.008, 95% CI: 0.003–0.020, *p* < 0.001, respectively). In contrast, the VHp score was significantly higher during the “under anesthesia, supine” period compared with the “awake, supine” period (OR = 5.716, 95% CI: 2.496–13.092, *p* < 0.001) (Table 6). At both Tsupine and Tpostop time points, a majority of lenses were categorized as well-opened (VHp score: 4; Tsupine: 39/40 [98%], Tpostop: 49/53 [92%]) (Table 5). Extreme narrowing of the anterior chamber (ICA <15°) was documented for two eyes across three time points (3/410 [1%]), with all episodes occurring during STP (Table 3). Changes in both ICA values and VHp scores during STP relative to Tpre for each eye are illustrated in Table 7. In over 60% of the examined eyes, the anterior chamber appeared narrower in the STP than in the preoperative state. Extreme anterior chamber narrowing (ICA < 15°) during STP occurred in one patient with a preoperative ICA of >30° and another patient with an ICA of 20–30°.

**Figure 3:**
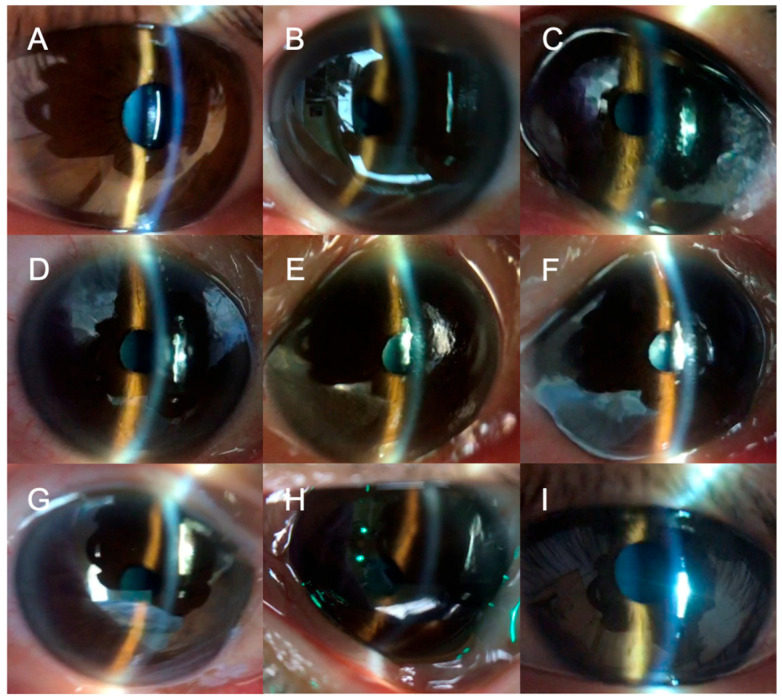
Photographs of the anterior segment of the right eye of a 57-year-old male, in a representative case at each time point. Significant narrowing of anterior chamber depth is seen during RALP. At Tpre, the anterior chamber appeared deep with dilated pupils (**A**). Following anesthesia induction, the anterior chamber maintained its width at Tanesth (**B**) but narrowed during STP at T0 and T1 (**C**,**D**) and displayed extreme narrowing at T2 and T3 (**E**,**F**). Post-return to the supine position (Tsupine and Tpostop), the narrowed anterior chamber deepened again (**G**,**H**, respectively). Next-day images in the awake supine position (**I**). During general anesthesia, the pupils were uniformly constricted.

Figure 3 illustrates a significant narrowing of the anterior eye morphology during RALP in the right eye in a 57-year-old male, with a noticeable narrowing observed during STP and further extreme narrowing at T2 and T3, followed by recovery after returning to the supine position.

The relationship between factors associated with intraoperative IOP elevation and narrowing of the ICA (ICA < 20°) at T2 was examined (Table 8) [6,8]. Univariate analysis indicated that only ETCO_2_ at T2 was associated with various factors (age, ICA, MAP, ETCO_2_, PIP, or infusion volume). Intraoperative factors one hour prior (T1), including (ICA, MAP, ETCO_2_, PIP and infusion volume), failed to predict ICA < 20° at T2.

## 4. Discussion

This study marks the first prospective study to evaluate anterior segment morphological changes during RALP. Our findings demonstrated a significant reduction in the ACD during STP, an occurrence noted in the majority of eyes, with two cases exhibiting pronounced changes. This anterior chamber configuration may serve as an anatomical risk factor for significant IOP elevation during RALP, potentially contributing to the development of postoperative ocular complications.

Rabinowitz et al. reported the incidence of ophthalmic injuries during RALP in 100,872 patients, with low rates of corneal abrasions, dry eye, retinal vascular occlusion, and ischemic optic neuropathy [33]. However, previous research regarding the presence of visual field defects induced by RALP has been conducted up to 6 months after surgery, and the long-term prevalence of perimetric abnormalities following RALP remains to be elucidated [7,10,13,14,15,16]. Visual field defects arising from IOP elevation may develop insidiously, often remaining undetected. The integrity of the delicate ocular structures is dependent upon IOP regulation; when IOP increases, ocular perfusion pressure exhibits an inverse linear correlation, potentially compromising retinal function, and the lower limits of autoregulation are approached when IOP reaches 30–35 mmHg [34,35,36]. IOP increase during RALP has been reported to be approximately 10 mmHg higher than preoperative measurements [7,9,10,12,16], and this increase is not enough to cause a disturbance in ocular biomechanical homeostasis [7,10,13,14,15,16]. However, numerous accounts of transient IOP exceeding 40 mmHg during RALP in healthy eyes have emerged [8,9]. Blecha et al. reported a case of IOP elevation reaching 59.6 mmHg during RALP [8]. Such extreme elevations are likely sufficient to induce postoperative visual loss. With an increasing number of RALP procedures executed globally, it is crucial to identify extreme IOP elevations occurring during RALP and develop strategies aimed at preventing postoperative visual field abnormalities [1,2,3,4,5].

An important mechanism underlying elevated IOP during RALP involves choroidal expansion due to both CO_2_ pneumoperitoneum and STP, with STP reported to have a greater effect [7,8,9]. Mondzelewski et al. indicated that CO_2_ pneumoperitoneum increased IOP by 3 mmHg, and the addition of STP contributed an additional increase of 11 mmHg [13]. The fluid shift toward the upper body due to STP substantially elevates IOP. The characteristics of the space environment associated with fluid shifts and elevated CO_2_ concentrations are similar to those encountered during RALP. Visual field abnormalities induced in astronauts during spaceflight have been referred to as spaceflight-associated neuro-ocular syndrome [37]. Experimental studies employing head-down tilt (HDT) have investigated the effects of reduced gravity on eyes at ground level, informing the development of new numerical models of the eye that incorporate changes in ocular hemodynamics, gravity, and intracranial pressure [38]. In HDT, the steeper the angle, the greater the IOP increase, and the IOP increase at 24° corresponding to RALP has been reported to be 3.6 mmHg [39,40]. This IOP increase is considerably less than that during RALP.

Induction agents, volatile anesthetics, muscle relaxants, and opioids used during general anesthesia decrease IOP [34]. Notably, IOP during RALP has been recorded to decrease by 3–11 mmHg following anesthesia induction [7,8,10,11,17]. Relaxation of the external ocular muscles due to sedation results in pupil constriction, morphologically enhancing aqueous humor outflow. In this study, pupil diameters were equally constricted under routine general anesthesia, and the ICA values at Tpostop, the VHp scores at Tsupine and Tpostop were higher than those at Tpre. Although it is known that IOP is less elevated under general anesthesia compared with that in conscious states, the pronounced elevation in IOP during RALP compared with HDT experiment results indicates additional factors contributing to substantial IOP increases that counteract the IOP-lowering effects of general anesthesia [41].

In HDT experiments, IOP reaches a new equilibrium state after approximately 1 h of application, remaining nearly constant thereafter [39]. Conversely, during RALP, a progressive increase in IOP continues throughout the duration of STP [7,8,9,10,12,16,17,18,19,20]. In RALP, a substantial volume of intravenous fluids is administered intraoperatively to mitigate the decrease in blood pressure attributable to general anesthesia, bleeding, and other factors. Edematous changes were observed in many eyes in this study (Supplementary Figure S2). Increases in circulating plasma volume as well as permeability lead to choroidal swelling, suggesting that intraoperative infusions may also contribute to IOP elevation during RALP as previously reported [7,11]. Nevertheless, the extreme IOP elevations observed in a subset of patients during RALP cannot be entirely attributed to infusion because intraoperative infusions are administered to all patients.

Position changes are known to alter anterior chamber morphology, often causing rapid IOP fluctuations. Previous studies have shown that head-down positioning shifts the iris and lens diaphragm anteriorly, redistributing aqueous humor and resulting in a shallower anterior chamber [42]. However, quantifying these changes remains challenging, and limitations exist within current acute gravity response numerical models concerning IOP from HDT research; notably, they fail to account for anterior chamber volume [38]. In this study, despite pupil constriction, quantitative analysis of the anterior segment during RALP demonstrated that >60% of the examined eyes exhibited significant shallowing in STP. Furthermore, pronounced anterior chamber narrowing was observed in two eyes. Under general anesthesia, the ICA/PD:CD ratio was significantly lower in STP than in the supine position. Morphological changes in the anterior chamber due to STP may be greater under general anesthesia than under awake conditions. Thickening of the uveal tissues, due to intraoperative hemorrhage or transfusion, could further contribute to anterior chamber shallowing. The degree of anterior chamber narrowing during RALP may be related to the degree of intraoperative IOP elevation.

No significant relationship was identified between intraoperative factors (MAP, PIP and infusion volume) and anterior chamber narrowing. Intraoperative factors one hour prior, including ICA, also failed to predict narrowing of the anterior chamber. A causal relationship between ETCO_2_ levels and anterior chamber shallowing was established, although the limited number of cases indicates a need for additional studies to further explore intraoperative factors affecting anterior chamber morphology. Presently, the degree of morphological changes occurring in the anterior chamber during RALP appears to vary among individuals. Eyes with inherently shallow anterior chambers, lens hypertrophy or anterior shift, and arcuate iris protrusions may display more pronounced anterior chamber narrowing during STP [43]. Elderly patients undergoing PCa surgery often have cataracts, which can cause lens enlargement and anterior chamber shallowing, leading to intraoperative narrowing. However, extreme anterior chamber narrowing during RALP is unpredictable preoperatively. In this study, both eyes with marked narrowing intraoperatively had sufficiently wide chambers preoperatively. Various methodologies aimed at IOP reduction during RALP have been reported, and these interventions can be employed when extreme elevations in IOP are detected [44]. The SEC enables easy acquisition of anterior segment images even in the STP (Supplementary Figure S3). Conventional ophthalmic imaging devices, such as anterior segment optical coherence tomography, are generally large and stationary, making them unsuitable for objectively assessing anterior segment morphological changes in the operating room. In contrast, the SEC is compact, portable, and easy to operate even by non-ophthalmologists, allowing convenient use in the operating room to obtain objective imaging records. Moreover, its ability to calculate ACD enables integration of quantitative measurements, facilitating a more objective assessment and potentially aiding in the prediction of IOP elevation during RALP [27,28].

This study has certain limitations. As a pilot investigation, the sample size was relatively small. Furthermore, the study exclusively recruited Japanese patients, and given that Asians reportedly demonstrate a higher prevalence of shallow anterior chambers affected by position changes, results may not be generalizable to other populations [45]. All anterior segment photographs were obtained under standard indoor lighting conditions. Although the images were taken in different settings (preoperatively in the urology clinic, intraoperatively in the operating room, and postoperatively in the ward), the ambient lighting levels were comparable and did not differ sufficiently to cause substantial variation in pupil size or configuration. Next, edema that developed during RALP impeded anterior segment observation via the SEC, as conjunctival edema occurred in numerous eyes intraoperatively (Supplementary Figure S2), thereby hindering adequate visualization of the anterior segment. Additionally, internet connectivity limitations within the operating room made it difficult to send videos. We used an iPhone 7; advancements in newer iPhone models and improvements in internet connectivity may address these limitations in future research. Also, it is imperative to note the absence of IOP measurements in this pilot study. Consequently, it remains uncertain if intraoperative anterior chamber narrowing directly correlates with significant IOP elevations. During RALP, the marked shallowing of the anterior chamber observed in STP may contribute to impaired aqueous humor outflow. Additionally, increased venous pressure and elevated CO_2_ levels caused by both CO_2_ pneumoperitoneum and STP coexist in this surgical setting. Even though IOP was not measured in this study, these factors collectively may lead to a rapid IOP elevation and a subsequent reduction in ocular perfusion pressure. Such mechanisms highlight the potential physiological significance of the anatomical narrowing of the anterior chamber angle observed during STP. To further elucidate the physiological significance of anterior chamber changes observed under STP, additional studies using larger samples incorporating simultaneous IOP measurement are warranted. Finally, this study does not directly compare the Smart Eye Camera with other standard intraoperative imaging devices, and this lack of head-to-head validation represents a methodological limitation. Device-based interpretation is further limited by the absence of concurrent IOP measurements in this setting, which should be considered when interpreting the findings.

## 5. Conclusions

Morphological changes in the anterior segment during RALP were evaluated. Despite pupil constriction associated with general anesthesia, the anterior chamber was narrowed during STP in numerous cases. Notably, a high degree of anterior chamber shallowing, which could potentially induce extreme IOP elevations, was documented in two eyes. By using the SEC, morphological changes in the anterior chamber can be observed in real time, providing the potential to identify unacceptable IOP elevations during RALP in the future.

## 6. Patents

OUI Inc. has the patent for the Smart Eye Camera and relations (Patent No. JP; 6627071, USA; 12,193,745, EU; 19743494.7, China; 201980010174-7, India; 541687, VN; 1-2020-04893, and Africa; AP6569, AP6839. Patent pending JP; 2019-198354, 2021-535472, 2022-500489, 2024-205339. EU; 20846667-2, 2175926.2, US; 17/799043, VN; 1-2022-01003, Africa; AP/P/2022/013851). There are no other relevant declarations relating to this patent.

## Figures and Tables

**Table 1:** Characteristics and surgical details of the study participants with prostate cancer.

Characteristics	Total (*N* = 29)
Age (years)	69.6 ± 6.1 (57–79)
Height (m)	1.68 ± 0.06 (1.59–1.81)
Weight (kg)	66.6 ± 10.2 (46.0–89.0)
BMI (kg/m^2^)	23.5 ± 2.8 (17.6–28.9)
Ocular disease	
None	26 (89.7%)
Normal tension open-angle glaucoma (bilateral)	3 (10.3%)
Anesthesia duration (min)	341.0 ± 86.7 (172–494)
Operation duration (min)	262.0 ± 81.8 (114–369)
Pneumoperitoneum duration (min)	224.4 ± 75.2 (98–337)
STP duration (min)	215.3 ± 72.9 (88–316)
Console duration (min)	199.8 ± 70.7 (79–294)
Infusion volume (mL)	2881.0 ± 871.5 (1450–5500)
Blood loss (mL)	336.4 ± 293.7 (10–1200)

BMI, body mass index; STP, steep Trendelenburg position.

**Table 2:** Mean ICA values, mean VHp score, and mean PD:CD ratio at various time points.

Time Point	Mean ICA (±SD)	Mean VHp Score (±SD)	Mean PD:CD Ratio (±SD)
Tpre	33.1 ± 4.0	3.70 ± 0.46	0.27 ± 0.04
Tanesth	34.1 ± 4.3	3.81 ± 0.40	0.16 ± 0.02
T0	28.4 ± 5.1	3.22 ± 0.51	0.16 ± 0.02
T1	27.7 ± 5.5	3.11 ± 0.64	0.17 ± 0.03
T2	27.4 ± 5.3	3.14 ± 0.69	0.17 ± 0.02
T3	26.8 ± 5.4	3.16 ± 0.60	0.18 ± 0.02
Tsupine	34.3 ± 4.5	3.98 ± 0.16	0.17 ± 0.02
Tpostop	35.0 ± 4.2	3.92 ± 0.27	0.18 ± 0.03
Tpost	32.8 ± 3.9	3.68 ± 0.47	0.26 ± 0.04

ICA, iridocorneal angle; VHp Score, Van Herick Plus’ score; PD:CD ratio, pupil diameter to corneal diameter ratio; SD, standard deviation; Tpre, time point preoperative; Tanesth, time point after anesthesia induction; T0, time point 0; T1 to T5, time points 1 to 5; Tsupine, time point supine; Tpostop, time point postoperative; Tpost, time point postoperative day.

**Table 3:** ICA values recorded at each measurement time point for all eyes.

		ICA			
Time Point	*N*	>30°	20–30°	15–20°	<15°
Tpre	57	45 (79%)	12 (21%)	0 (0%)	0 (0%)
Tanesth	47	38 (81%)	9 (19%)	0 (0%)	0 (0%)
T0	49	13 (27%)	35 (71%)	1 (2%)	0 (0%)
T1	53	16 (30%)	32 (60%)	4 (8%)	1 (2%)
T2	35	11 (31%)	21 (60%)	2 (6%)	1 (3%)
T3	19	6 (32%)	11 (58%)	1 (5%)	1 (5%)
Tsupine	40	31 (78%)	9 (23%)	0 (0%)	0 (0%)
Tpostop	53	45 (85%)	8 (15%)	0 (0%)	0 (0%)
Tpost	57	42 (74%)	15 (26%)	0 (0%)	0 (0%)
Awake, supine	114	87 (76%)	27 (24%)	0 (0%)	0 (0%)
Under anesthesia, supine	140	114 (81%)	26 (19%)	0 (0%)	0 (0%)
Under anesthesia, STP	156	46 (29%)	99 (63%)	8 (5%)	3 (2%)

**Table 4:** Comparison of ICA values under different conditions: Analysis using an ordinal logistic mixed model.

	ICA					
	OR	95% CI	*p* Value	OR	95% CI	*p* Value
Awake, supine (Tpre, Tpost)	1.000	Ref				
Under anesthesia, supine (Tanesth, Tsupine, Tpostop)	1.534	0.749, 3.141	0.241	1.000	ref	
Under anesthesia, STP (T0–T3)	0.050	0.025, 0.101	<0.001	0.033	0.016, 0.067	<0.001

OR: Odds ratio; 95% CI: 95% confidence interval; ref: reference category.

**Table 5:** VHp scores recorded at each measurement time point for all eyes.

		VHp Score			
Time Point	*N*	4	3	2	1
Tpre	57	40 (70%)	17 (30%)	0 (0%)	0 (0%)
Tanesth	47	38 (81%)	9 (19%)	0 (0%)	0 (0%)
T0	49	13 (27%)	34 (69%)	2 (4%)	0 (0%)
T1	53	14 (26%)	31 (58%)	8 (15%)	0 (0%)
T2	35	11 (31%)	18 (51%)	6 (17%)	0 (0%)
T3	19	5 (26%)	12 (63%)	2 (11%)	0 (0%)
Tsupine	40	39 (98%)	1 (3%)	0 (0%)	0 (0%)
Tpostop	53	49 (92%)	4 (8%)	0 (0%)	0 (0%)
Tpost	57	39 (68%)	18 (32%)	0 (0%)	0 (0%)
Awake, supine	114	79 (69%)	35 (31%)	0 (0%)	0 (0%)
Under anesthesia, supine	140	126 (90%)	14 (10%)	0 (0%)	0 (0%)
Under anesthesia, STP	156	43 (28%)	95 (61%)	18 (12%)	0 (0%)

**Table 6:** Comparison of VHp score under different conditions: Analysis using an ordinal logistic mixed model.

	ICA					
	OR	95% CI	*p* Value	OR	95% CI	*p* Value
Awake, supine (Tpre, Tpost)	1.000	ref				
Under anesthesia, supine (Tanesth, Tsupine, Tpostop)	5.716	2.496, 13.092	<0.001	1.000	ref	
Under anesthesia, STP (T0–T3)	0.048	0.023, 0.100	<0.001	0.008	0.003, 0.020	<0.001

**Table 7:** Change in ICA values and VHp scores compared with Tpre for each eye.

ICA (Tpre)	Minimum ICA Value in STP	*N* (%)	VHp Score (Tpre)	Minimum VHp Score in STP	*N* (%)
>30° (45 eyes)	>30°	11 (19%)	4 (40 eyes)	4	8 (14%)
	20–30°	30 (53%)		3	28 (49%)
	15–20°	3 (5%)		2	4 (7%)
	<15°	1 (2%)			
20–30° (12 eyes)	>30°	0 (0%)	3 (17 eyes)	3	12 (21%)
	20–30°	10 (18%)		2	5 (9%)
	15–20°	1 (2%)			
	<15°	1 (2%)			
Total (57 eyes)	No change	21 (37%)	Total (57 eyes)	No change	20 (35%)
	Shallow change	36 (63%)		Shallow change	37 (65%)

**Table 8:** A Study of Predictive Factors for ICA < 20° at T2.

ICA < 20 at T2: *N* = 3			Univariate			
			OR	95% CI		*p* Value
Age		per 1	0.916	0.757	1.110	0.361
ICA	T1	per 1	0.809	0.643	1.019	0.070
MAP (mmHg)	T1	per 1	1.014	0.893	1.152	0.823
	T2	per 1	1.000	0.892	1.122	0.999
ETCO_2_ (mmHg)	T1	per 1	1.556	1.000	2.421	0.050
	T2	per 1	1.697	1.026	2.807	0.040
PIP (cmH_2_O)	T1	per 1	0.955	0.618	1.477	0.832
	T2	per 1	0.929	0.642	1.346	0.690
Infusion volume (mL)	T1	per 1	1.000	0.998	1.002	0.728
	T2	per 1	1.000	0.998	1.002	0.862

MAP, mean arterial pressure; ETCO_2_, end tidal CO_2_; PIP, peak inspiratory pressure.

## Data Availability

The data used and analyzed for this study are available from the corresponding author on reasonable request.

## Supplementary Materials

The following supporting information can be downloaded at https://www.mdpi.com/article/10.3390/jcm15020731/s1. Supplementary Figure S1: Selection of video images for analysis; Supplementary Figure S2: Subcutaneous edema of the conjunctiva; Supplementary Figure S3: Video recording of anterior segment with Smart Eye Camera; Supplementary Table S1: Inter-rater reliability of the VHp score; Supplementary Table S2: Inter-rater reliability of ICA/PD:CD ratio.

## Abbreviations

The following abbreviations are used in this manuscript:

ACD	Anterior Chamber Depth
CI	Confidence Interval
CD	Corneal Diameter
CO_2_	Carbon Dioxide
CVP	Central Venous Pressure
ETCO_2_	End-Tidal Carbon Dioxide
HDT	Head-Down Tilt
ICA	Iridocorneal Angle
ICCs	Intraclass Correlation Coefficients
IOP	Intraocular Pressure
MAP	Mean Arterial Pressure
PaCO_2_	Partial Pressure of Carbon Dioxide
PAC	Peripheral Anterior Chamber
PCa	Prostate Cancer
PCT	Peripheral Corneal Thickness
PD	Pupil Diameter
PIP	Peak Inspiratory Pressure
RALP	Robot-Assisted Laparoscopic Prostatectomy
SD	Standard Deviation
SEC	Smart Eye Camera
STP	Steep Trendelenburg Position
VHp	Van Herick Plus

## References

[1] Bray F., Laversanne M., Sung H., Ferlay J., Siegel R.L., Soerjomataram I., Jemal A. (2024). Global cancer statistics 2022: GLOBOCAN estimates of incidence and mortality worldwide for 36 cancers in 185 countries. CA Cancer J. Clin..

[2] Droz J.P., Albrand G., Gillessen S., Hughes S., Mottet N., Oudard S., Payne H., Puts M., Zulian G., Balducci L. (2017). Management of Prostate Cancer in Elderly Patients: Recommendations of a Task Force of the International Society of Geriatric Oncology. Eur. Urol..

[3] Schafer F.J., Laversanne M., Sung H., Soerjomataram I., Briganti A., Dahut W., Bray F., Jemal A. (2025). Recent Patterns and Trends in Global Prostate Cancer Incidence and Mortality: An Update. Eur. Urol..

[4] Gray P.J., Lin C.C., Cooperberg M.R., Jemal A., Efstathiou J.A. (2017). Temporal Trends and the Impact of Race, Insurance, and Socioeconomic Status in the Management of Localized Prostate Cancer. Eur. Urol..

[5] Okhawere K.E., Shih I.F., Lee S.H., Li Y., Wong J.A., Badani K.K. (2021). Comparison of 1-Year Health Care Costs and Use Associated with Open vs Robotic-Assisted Radical Prostatectomy. JAMA Netw. Open.

[6] Mendel E., Stoicea N., Rao R. (2017). Revisiting Postoperative Vision Loss following Non-Ocular Surgery: A Short Review of Etiology and Legal Considerations. Front. Surg..

[7] Taketani Y., Mayama C., Suzuki N. (2015). Transient but significant visual field defects after robot-assisted laparoscopic radical prostatectomy in deep tRendelenburg position. PLoS ONE.

[8] Blecha S., Harth M., Schlachetzki F., Zeman F., Blecha C., Flora P., Burger M., Denzinger S., Graf B.M., Helbig H. (2017). Changes in intraocular pressure and optic nerve sheath diameter in patients undergoing robotic-assisted laparoscopic prostatectomy in steep 45° Trendelenburg position. BMC Anesthesiol..

[9] Rosendal C., Markin S., Hien M.D., Motsch J., Roggenbach J. (2014). Cardiac and hemodynamic consequences during capnoperitoneum and steep Trendelenburg positioning: Lessons learned from robot-assisted laparoscopic prostatectomy. J. Clin. Anesth..

[10] Awad H., Santilli S., Ohr M., Roth A., Yan W., Fernandez S., Roth S., Patel V. (2009). The effects of steep trendelenburg positioning on intraocular pressure during robotic radical prostatectomy. Anesth. Analg..

[11] Hoshikawa Y., Tsutsumi N., Ohkoshi K., Serizawa S., Hamada M., Inagaki K., Tsuzuki K., Koshimizu J., Echizen N., Fujitani S. (2014). The effect of steep Trendelenburg positioning on intraocular pressure and visual function during robotic-assisted radical prostatectomy. Br. J. Ophthalmol..

[12] Raz O., Boesel T.W., Arianayagam M., Lau H., Vass J., Huynh C.C., Graham S.L., Varol C. (2015). The effect of the modified Z trendelenburg position on intraocular pressure during robotic assisted laparoscopic radical prostatectomy: A randomized, controlled study. J. Urol..

[13] Mondzelewski T.J., Schmitz J.W., Christman M.S., Davis K.D., Lujan E., L’Esperance J.O., Auge B.K. (2015). Intraocular Pressure During Robotic-assisted Laparoscopic Procedures Utilizing Steep Trendelenburg Positioning. J. Glaucoma.

[14] Kakutani S., Asamoto M., Araki F., Chen Y.N., Shinokawa M., Okagami Y., Ohata T., Taguchi S., Yamada Y., Takeshima Y. (2020). Prospective evaluation of visual function in patients with ocular diseases after robot-assisted laparoscopic prostatectomy. Int. J. Urol..

[15] Hirooka K., Ukegawa K., Nitta E., Ueda N., Hayashida Y., Hirama H., Taoka R., Sakura Y., Yamasaki M., Tsunemori H. (2018). The Effect of Steep Trendelenburg Positioning on Retinal Structure and Function during Robotic-Assisted Laparoscopic Procedures. J. Ophthalmol..

[16] Awad H., Bai M., Ramadan M.E., Shabsigh A., Backes F., Craven M.A., Abdel-Rasoul M., Bergese S.D., Slabaugh M. (2020). The Effect of Increased Intraocular Pressure During Steep Trendelenburg Positioning in Robotic Prostatectomy and Hysterectomy on Structural and Functional Ocular Parameters. Anesth. Analg..

[17] Shirono Y., Takizawa I., Kasahara T., Maruyama R., Yamana K., Tanikawa T., Hara N., Sakaue Y., Togano T., Nishiyama T. (2020). Intraoperative intraocular pressure changes during robot-assisted radical prostatectomy: Associations with perioperative and clinicopathological factors. BMC Urol..

[18] Yoo Y.C., Kim N.Y., Shin S., Choi Y.D., Hong J.H., Kim C.Y., Park H., Bai S.J. (2015). The Intraocular Pressure under Deep versus Moderate Neuromuscular Blockade during Low-Pressure Robot Assisted Laparoscopic Radical Prostatectomy in a Randomized Trial. PLoS ONE.

[19] Yoo Y.C., Shin S., Choi E.K., Kim C.Y., Choi Y.D., Bai S.J. (2014). Increase in intraocular pressure is less with propofol than with sevoflurane during laparoscopic surgery in the steep Trendelenburg position. Can. J. Anaesth..

[20] Kim N.Y., Yoo Y.C., Park H., Choi Y.D., Kim C.Y., Bai S.J. (2015). The effect of dexmedetomidine on intraocular pressure increase in patients during robot-assisted laparoscopic radical prostatectomy in the steep Trendelenburg position. J. Endourol..

[21] Ozcan M.F., Akbulut Z., Gurdal C., Tan S., Yildiz Y., Bayraktar S., Ozcan A.N., Ener K., Altinova S., Arslan M.E. (2017). Does steep Trendelenburg positioning effect the ocular hemodynamics and intraocular pressure in patients undergoing robotic cystectomy and robotic prostatectomy?. Int. Urol. Nephrol..

[22] Weber E.D., Colyer M.H., Lesser R.L., Subramanian P.S. (2007). Posterior ischemic optic neuropathy after minimally invasive prostatectomy. J. Neuro-Ophthalmol..

[23] Bonomi L., Marchini G., Marraffa M., Bernardi P., De Franco I., Perfetti S., Varotto A. (2000). Epidemiology of angle-closure glaucoma: Prevalence, clinical types, and association with peripheral anterior chamber depth in the Egna-Neumarket Glaucoma Study. Ophthalmology.

[24] Wu S.C., Lee Y.S., Wu W.C., Chang S.H. (2016). Chamber depth and angle-closure glaucoma after central retinal vein occlusion. BMC Ophthalmol..

[25] Roor T.L., Kooijman J.A., van der Ploeg J.M., de Boer H.D. (2019). Postoperative Acute Angle-Closure Glaucoma: A Rare but Serious Complication: A Case Report. A A Pract..

[26] Shimizu E., Yazu H., Aketa N., Yokoiwa R., Sato S., Katayama T., Hanyuda A., Sato Y., Ogawa Y., Tsubota K. (2021). Smart Eye Camera: A Validation Study for Evaluating the Tear Film Breakup Time in Human Subjects. Transl. Vis. Sci. Technol..

[27] Shimizu E., Yazu H., Aketa N., Yokoiwa R., Sato S., Yajima J., Katayama T., Sato R., Tanji M., Sato Y. (2021). A Study Validating the Estimation of Anterior Chamber Depth and Iridocorneal Angle with Portable and Non-Portable Slit-Lamp Microscopy. Sensors.

[28] Mizukami T., Shimizu E., Tanaka K., Nishimura H., Nakayama S., Yokoiwa R., Ueno S., Mishima S., Shimomura Y. (2025). Validation of an Artificial Intelligence-Based Anterior Chamber Depth Estimation Using a Smartphone-Compatible Slit Lamp Device. Ophthalmol. Sci..

[29] Li W., Chen Q., Jiang C., Shi G., Deng G., Sun X. (2021). Automatic Anterior Chamber Angle Classification Using Deep Learning System and Anterior Segment Optical Coherence Tomography Images. Transl. Vis. Sci. Technol..

[30] Kim S.H., Kang J.H., Park K.H., Hong C. (2012). Hong’s grading for evaluating anterior chamber angle width. Jpn. J. Ophthalmol..

[31] Călugăru D., Călugăru M. (2021). Intraocular pressure modifications in patients with acute central/hemicentral retinal vein occlusions. Int. J. Ophthalmol..

[32] Sihota R., Kamble N., Sharma A.K., Bhari A., Gupta A., Midha N., Selvan H., Dada T., Gupta V., Pandey R.M. (2019). ‘Van Herick Plus’: A modified grading scheme for the assessment of peripheral anterior chamber depth and angle. Br. J. Ophthalmol..

[33] Rabinowitz J., Kinnear N., O’Callaghan M., Hennessey D., Shafi F., Fuller A., Ibrahim M., Lane T., Adshead J., Vasdev N. (2024). Systematic review of the ophthalmic complications of robotic-assisted laparoscopic prostatectomy. J. Robot. Surg..

[34] Kelly D.J., Farrell S.M. (2018). Physiology and Role of Intraocular Pressure in Contemporary Anesthesia. Anesth. Analg..

[35] Bansal A.S., Hsu J., Garg S.J., Sivalingam A., Vander J.F., Moster M., Maguire J.I., Regillo C.D. (2012). Optic neuropathy after vitrectomy for retinal detachment: Clinical features and analysis of risk factors. Ophthalmology.

[36] Hayreh S.S. (2001). Blood flow in the optic nerve head and factors that may influence it. Prog. Retin. Eye Res..

[37] Lawley J.S., Petersen L.G., Howden E.J., Sarma S., Cornwell W.K., Zhang R., Whitworth L.A., Williams M.A., Levine B.D. (2017). Effect of gravity and microgravity on intracranial pressure. J. Physiol..

[38] Nelson E.S., Mulugeta L., Feola A., Raykin J., Myers J.G., Samuels B.C., Ethier C.R. (2017). The impact of ocular hemodynamics and intracranial pressure on intraocular pressure during acute gravitational changes. J. Appl. Physiol..

[39] Nelson E.S., Myers J.G., Lewandowski B.E., Ethier C.R., Samuels B.C. (2020). Acute effects of posture on intraocular pressure. PLoS ONE.

[40] Marshall-Goebel K., Mulder E., Bershad E., Laing C., Eklund A., Malm J., Stern C., Rittweger J. (2017). Intracranial and Intraocular Pressure During Various Degrees of Head-Down Tilt. Aerosp. Med. Hum. Perform..

[41] Sator-Katzenschlager S., Deusch E., Dolezal S., Michalek-Sauberer A., Grubmüller R., Heinze G., Wedrich A. (2002). Sevoflurane and propofol decrease intraocular pressure equally during non-ophthalmic surgery and recovery. Br. J. Anaesth..

[42] Rakova N., Jüttner K., Dahlmann A., Schröder A., Linz P., Kopp C., Rauh M., Goller U., Beck L., Agureev A. (2013). Long-term space flight simulation reveals infradian rhythmicity in human Na(+) balance. Cell Metab..

[43] Huang G., Gonzalez E., Peng P.H., Lee R., Leeungurasatien T., He M., Porco T., Lin S.C. (2011). Anterior chamber depth, iridocorneal angle width, and intraocular pressure changes after phacoemulsification: Narrow vs open iridocorneal angles. Arch. Ophthalmol..

[44] Ackerman R.S., Cohen J.B., Getting R.E.G., Patel S.Y. (2019). Are you seeing this: The impact of steep Trendelenburg position during robot-assisted laparoscopic radical prostatectomy on intraocular pressure: A brief review of the literature. J. Robot. Surg..

[45] Schuster A.K., Pfeiffer N., Nickels S. (2016). Distribution of Anterior Chamber Angle Width and Correlation with Age, Refraction, and Anterior Chamber Depth-The Gutenberg Health Study. Investig. Ophthalmol. Vis. Sci..

