# Supplementary material for: Prospective Evaluation of Ocular Anterior Segment Morphology Changes in the Steep Trendelenburg Position During Robotic-Assisted Laparoscopic Prostatectomy
Source: J Clin Med. 2026 Jan 16;15(2):731. doi: 10.3390/jcm15020731 (PMC12841824; doi:10.3390/jcm15020731)
Supplement: Supplementary file 1 [file jcm-15-00731-s001.zip › jcm-4098255-supplementary.pdf]

Supplementary Table S1. Inter-rater reliability of the VHp score.

n		Evaluator: S.K.		
		2	3	4
Evaluator: E.S.	2	225	124	0
	3	50	3232	716
	4	0	151	8416

Kappa statistic: 0.819 [95% CI: 0.809 - 0.830]

VHp Score, Van Herick Plus' score; CI, confidence interval

Supplementary Table S2. Inter-rater reliability of ICA/PD:CD ratio.

	ICC(2,1)	95%CI
ICA	0.931	0.929 - 0.933
PD:CD ratio	0.946	0.944 - 0.948

ICC (2, 1): Intraclass Correlation Coefficient with two-way random model
ICA, iridocorneal angle; PD:CD ratio, pupil diameter to corneal diameter ratio
ICC, Intraclass Correlation Coefficients

Supplementary Figure S1. Selection of video images for analysis

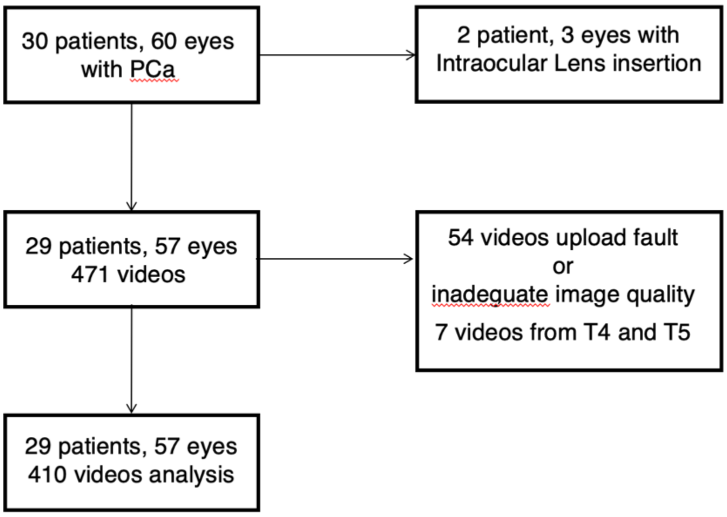

Flowchart showing the selection of video images for analysis. Among 30 patients (60 eyes), 3 eyes with intraocular lenses were excluded. Of 471 recorded videos, 61 were removed because of upload failures, inadequate image quality, or absence of T4/T5 recordings, resulting in 410 videos from 29 patients (57 eyes) included in the final analysis.

Supplementary Figure S2. Subcutaneous edema of the conjunctiva

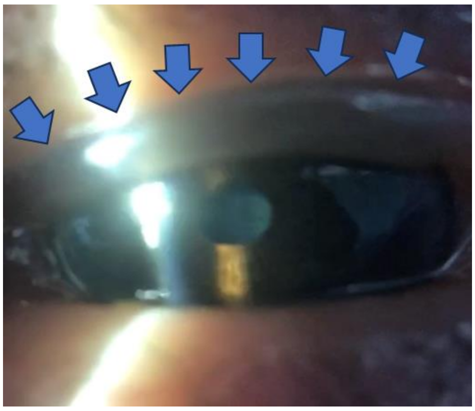

Conjunctival edema observed in the steep Trendelenburg position during robot-assisted laparoscopic prostatectomy, as indicated by the blue arrows.

Supplementary Figure S3. Video recording of anterior segment with Smart Eye Camera

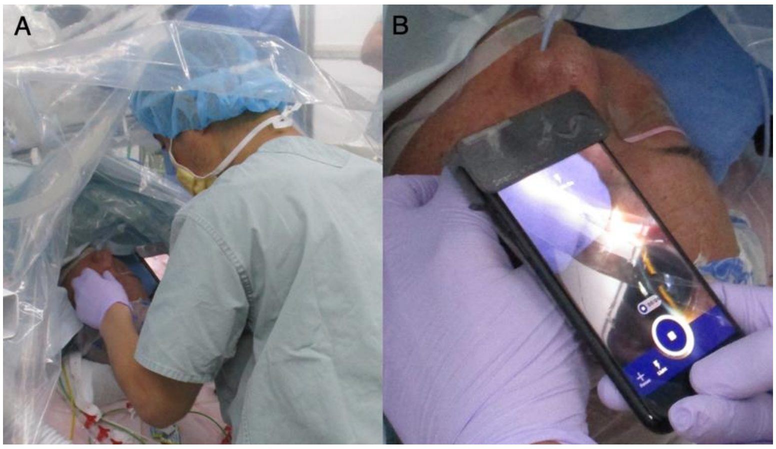

Video recording of the anterior segment using the Smart Eye Camera in the steep Trendelenburg position during robot-assisted laparoscopic prostatectomy. (A) External view of the examiner positioning the device. (B) The Smart Eye Camera placed beneath the surgical drape to capture the anterior segment.
